# Feasibility of lung cancer screening in low-risk populations of the Nordic countries

**DOI:** 10.2340/1651-226X.2026.45489

**Published:** 2026-03-18

**Authors:** Mef Nilbert, Gunnar Wagenius, Simon Ekman

**Affiliations:** a Department of Hematology, Pathology and Radiophysics, Skane University Hospital, Lund, Sweden; b Division of Oncology, Institute of Clinical Sciences, Lund University, Lund, Sweden; c Lung Oncology Center, Cancer Theme, Karolinska University Hospital, Stockholm, Sweden; d Department of Oncology and Pathology, Karolinska Institutet, Stockholm, Sweden; e Medical Unit for Head-Neck-Lung- Skin Cancer, Theme Cancer, Karolinska University Hospital, Stockholm, Sweden; f Department of Oncology-Pathology, Karolinska Institutet, Stockholm, Sweden

## Introduction

Lung cancer screening programmes represent a risk-based approach to early diagnosis of cancer aimed to optimise the balance between benefit and harm by targeting current and former smokers. The randomised controlled National Lung Screening Trial (NLST) and the NELSON trial showed lung cancer detection rates of approximately 1% and demonstrated around 20% reduced lung cancer mortality from screening of individuals at increased risk [1, 2]. Also the Italian randomised Multicentric Italian Lung Detection (MILD) screening trial demonstrated a long-term survival benefit of screening with low-dose computed tomography (LDCT) with a 39% risk reduction for lung cancer mortality at 10 years [3].These findings sparked interest and activity in lung cancer screening programmes and have led to a recommendation from the Council of the European Union to initiate pilot programmes targeted at individuals at increased risk [4].

LDCT has in several studies demonstrated high sensitivity for detection of small pulmonary nodules while minimising radiation exposure [5]. Advances in nodule management protocols, volumetric assessment, and risk stratification algorithms have substantially reduced false-positive rates and improved clinical efficiency. Nordic consensus statements have also been developed [6]. As with all screening programmes, lung cancer screening is associated with potential harms, such as false-positive findings, overdiagnosis of indolent tumours, radiation exposure, psychological distress and ethical considerations related to non-smoke related lung cancer [7].

Across the Nordic countries, lung cancer is the leading cause of cancer-related death with the majority of the cases being diagnosed at a late stage, which motivates an interest in early diagnostics and better outcomes through lung cancer screening. At the same time, daily tobacco smoking rates have declined significantly during recent decades to an average of 10%, ranging from 6% in Sweden to 15% in Denmark though smoking rates vary considerably between different groups in the population. Lung cancer shows a declining incidence among men and a stagnated incidence in women. This raises questions related to timing and feasibility of lung cancer screening in the Nordic countries since outreach to and participation from the appropriate target population is central to the effectiveness and cost-effectiveness of lung cancer screening programmes [8].

Feasibility and implementation of lung cancer screening with LDCT is being evaluated in a multitude of trials and some countries, e.g. UK, Czech Republic, Croatia, Hungary and Poland, have implemented national screening programmes. None of the Scandinavian countries have yet established a national lung cancer screening programme. Rather, various forms of pilot trials have been initiated and three of these, in Sweden, Norway and Finland, have recently reported their first lessons based on publications in Acta Oncologica [9–11]. In Denmark, a pilot study on lung cancer screening is currently underway in Southern Denmark, led by Odense University Hospital and commissioned by the Danish Health Authority. It includes current and former heavy smokers aged 60–74 living on Funen and nearby islands, who are invited to annual LDCT scans (Clinicaltrials.gov. NCT06537804).

Acta Oncologic has recently published results from lung cancer screening pilot studies in Sweden, Norway and Finland. Grundberg et al. report the results of the StockholmPLUS study [9]. The study included women age 54–74, current or former (within 10 years) smokers, with minimum 15 pack-years exposure. A smoking cessation programme was also included in the study. LDCT scans were evaluated by two board-certified radiologists who also had access to an evaluation based on the Artificial Intelligence (AI)-Rad Companion Chest Computed Tomography (CT) for automated detection and measurement of solid lung nodules. The trial developed a reporting system that categorised participants into five groups linked to distinct management pathways. In total, 34,580 individuals were invited, of which 11,607 (33.4%) completed the questionnaire. Of these, 10% met inclusion criteria and of these 90% underwent baseline LDCT. At baseline, 79% were classified as negative/benign and 6% were classified as suspected of cancer. Following evaluation 4.5% of the individuals were subject to diagnostic work-up and 1.3% were diagnosed with lung cancer, which increased to 1.5% after follow-up. The lung cancers were in 93% early-stage (stages I–II) tumours that could be treated with curative intent. The false positive rate was 0.2%. The AI-assisted model misclassified 3% of the cases and could reduce the workload by 49%. An extension of the study is now ongoing and invites both men and women and in addition another pilot study involving two more Swedish health care regions has been launched.

Mahovkic et al. report the baseline results from the Norwegian lung cancer screening pilot trial with the aim to explore feasibility and efficiency of a regional lung cancer screening programme [10]. A total of 125,095 individuals aged 60–79 years in one county were invited to participate in the study of which 10.6% consented to participate. Current smokers or former (within 10 years) smokers were invited to complete questionnaire based on the risk-prediction model PLCOm2012_NoRace._ Individuals with ≥ 35 pack-years or ≥ 2.6% or 6-year lung cancer risk were eligible for inclusion. All 2499 eligible participants were randomised to either screening or control group. In the intervention arm, 1006 individuals (79.5%) underwent baseline LDCT. Images were assessed by an experienced thoracic radiologist with possibility for consulting other senior thoracic radiologists. Screen-detected nodules were managed according to Lung-RADS v2022. At baseline, 83.9% of participants were classified as Lung-RADS 1–2 and were subsequently referred to the next screening round and 2.3% were classified as Lung-RADS 4B and were referred directly for radiological staging. In total, 2.8% were referred to pulmonologist evaluation. At baseline, lung cancer was diagnosed in 2.3%, of which 92% were stage I–II tumours.

Most cancers were detected at an early stage: 83% in stage I and 9% in stage II, while remaining 8% were in stage III (Table 3). In the study, 87% underwent thoracic surgery and 55% remained in same stage, while 35% were upstaged and 10% downstaged. Only one of 20 patients (5%) who underwent surgery had pathological stage III. One cancer case was overlooked at baseline. In total, 87% underwent robot-assisted thoracic surgery (RATS), one (4%) received stereotactic radiation due to poor lung function, and two received chemo-immunotherapy for locally advanced disease, whereof both passed away during the follow-up period. No other serious events were reported. By this definition, the false positive rate was 1.7% after radiological staging and 0.6% after clinical referral. In the Norwegian trial, the overall false positive rate, including all indeterminate and suspicious nodules was 11.4%, whereas the clinically relevant false positive rate was 1.7% after radiological staging and 0.6% after pulmonologist referral. Confirmed lung cancers were discussed at multidisciplinary tumour boards to determine treatment strategy. Most underwent curative treatment, primarily robot-assisted surgery.

Wichmann et al. report on the Finnish LDCT-SC-FI study that investigated variable recruitment strategies and smoking cessation methods in a randomised controlled sample of 200 individuals at increased risk age of 50–74 years [11, 12]. The participants were current or former smokers reporting minimum 30 pack-years. Study recruitment was by various means; newspaper, internet advertisements and informing relevant healthcare units at hospital district. Study participants were included at the Oulu University Hospital in Finland. Smoking cessation was randomised to be through a smartphone application or written material with higher rates for self-reported smoking cessation for patients randomised to the smartphone app. The study demonstrated that newspaper advertisements were the most effective recruitment method [11]. All study participants were offered LDCT screening at baseline and after 1 year with an uptake rate of 96.7% for both rounds combined. All scans were evaluated individually by three radiologists specialised in thoracic radiology and lesion volumes were assessed with the use of semiautomated software. Six lung cancers were detected, of which 5 was stage I, which corresponds to a positive predictive value of 75%. The lung cancer detection rate was 1.0% at baseline and increased to 2.2% at the second screening round. Of the cancer diagnosed, 83.3% were early-stage tumours and all of these underwent curative intent treatment.

Effective implementation of lung cancer screening requires outreach to the groups most likely to benefit from screening, high response rates, standardised protocols for image acquisition and nodule management, effective diagnostic processes, integration with smoking cessation interventions and optimised care pathways [13]. Studies that investigate eligibility criteria, participation rates, AI for risk prediction and image interpretation, and cost-effectiveness therefore provide valuable information to inform future lung cancer screening strategies. To further increase the quality and outcome of lung cancer screening programmes, it is also recommended to include risk models based on clinical factors, to use volumetric measurements for the assessment of growth rate and to include the study of biomarkers to enrich screening subgroups [14].

The Nordic lung cancer screening pilots are all designed for population-based recruitment and their first results suggest that lung cancer screening is feasible. However, the pilot studies have shortcomings and do not provide reliable data on efficacy due to limited sample sizes and single-centre design. Selection bias is likely to apply to all three studies with a risk for over-representation of health-conscious individuals, though the addition of a risk-prediction model that also take e.g. socioeconomic factors into account, in the Norwegian study counteracts this effect. The increasing need for diagnostic capacity needs to be considered. A recent population-based study from Denmark reported 186 pulmonary nodules/100.000 in the population [15]. Of these, 51.0% received a low-dose chest CT scan and 7.2% were referred for lung cancer diagnostic evaluation.

Screening programmes come with challenges that may be unique for each country and that require country-specific solutions. Each of these Nordic studies is an important contribution to the collective lessons from implementing lung cancer screening programmes in Europe. It is of vital importance that existing screening data be shared and analysed across countries to learn from successful approaches and to guide effective implementation. An example of such an initiative is the Strengthening the Screening of Lung Cancer in Europe (SOLACE) project, supported by Europe’s Beating Cancer Plan. This initiative brings together an extensive pan-European network of respiratory and radiology experts, developing targeted recruitment strategies for under-represented and high-risk populations [14]. Another EU-funded initiative is the CanScreen-European Cancer Information System (ECIS) project that aims to develop a new cancer screening data management system to be integrated into the existing ECIS (https://canscreen-ecis.iarc.who.int/). The aims are to improve the quality and completeness of data to be collected from different cancer screening programmes in Europe including lung cancer, to better monitor the effectiveness of these programmes. Organised cancer screening programmes are resource-intensive public health endeavours requiring a high degree of complex organisation and to support the implementation of lung cancer screening programmes, evidence-based pan-European technical standards have been published [16].

Whereas the Swedish and the Norwegian studies contacted individuals directly by mail, the Finnish study concluded that advertisements were the most efficient mode of contact. The Norwegian study showed a participation rate of 10.6%, whereas the Swedish study had a response rate of 33%. The difference could depend not only on contextual factors such as geographical area but also on the information provided since the Swedish study invited to participation, whereas the Norwegian study included a risk-prediction model and a randomisation step. This can be compared to participation rates of 25–30% in large international trials [1]. Participation rates were also somewhat higher in Sweden (90%) compared to Norway (79.5%). Several ongoing trials and national screening programmes identify smokers through medical records with screening inclusion based on referrals from family physicians. Studies have shown that preinvitation, invitation, and reminder letters from primary care physicians offering prescheduled appointments is associated with higher uptake rates [17]. These different strategies have pros and cons related to trustworthy and complete information. The optimal approach for the Nordic countries could potentially represent a mix of referrals and targeted identification of high-risk individuals.

Age profiles differed somewhat between the studies and were 50–74 in the Finnish study, 54–74 in the Swedish study and 60–79 in the Norwegian study. In the landmark NELSON and NLST trials, the age groups were 50–75 and 55–74, respectively [1]. The U.S. Preventive Services Task Force (USPSTF) recommends lung cancer screening in the age group of 50–80 [2]. Even though there are minor variations in age groups between the studies, they all aim to detect cancer at an early stage in high-risk, asymptomatic individuals. In previous screening studies males have been over-represented, whereas the Finnish study showed an equal sex distribution and the Swedish study provides female-specific data. In Denmark, the high-risk target population for lung cancer screening has been addressed using various eligibility criteria with demonstration of a lower sensitivity for women than for men. The observations of the feasibility of lung cancer screening in women in the Finnish and Swedish studies supports observations in subgroup analyses from e.g. the NELSON study [1, 9, 12].

The Swedish study had a single-found design whereas the Norwegian and Finnish studies included follow-up investigations with 2-year intervals and showed adherence to screening, which is crucial for a cancer screening programme to be effective [10, 12]. An AI-assisted algorithm was included in the LDCT evaluations in the Swedish study, which proved a significant reduction in workload. The Norwegian approach included a risk-prediction model that takes factors such as smoking intensity, duration, quit-time, family history, comorbidities, and socioeconomic background into accounts. This approach has been suggested to increase efficiency through broader identification of high-risk profiles. Several lung cancer risk-prediction models showed good performance in European countries and might improve the efficiency of lung cancer screening [18, 19]. These approaches with risk scores and AI-assisted imaging are in line with meeting the challenges identified in a Nordic survey-based study among respiratory physicians in Sweden, Finland, Norway, and Denmark to explore their views on LDCT screening. Major barriers identified included a lack of trained personnel and limited access to CT scanners, besides financial constraints [20].

Though the samples are limited, the Nordic studies showed lung cancer detection rates of 1.5–2.3%, which is comparable to the international trials that have shown 1–2% lung cancer detection rates. False positive findings compared favourable to current data and lung cancer diagnosed were in 83–87% stage I cancers, which indeed exceeded the rate detected in other international trials.

Integrated smoking cessation interventions are recommended since 7–23% of the individuals participating in LDCT programmes successfully quit smoking [21]. Nonetheless, the methods for smoking cessation in LDCT screening context are not well established. The Finnish study suggests that use of a smartphone application increases the chance for smoking cessation [12]. In one prospective randomised study, an integrated approach involving both medication and intensive counseling provided the optimal smoking cessation intervention in the lung cancer screening setting. A meta-analysis demonstrated that more intensive interventions (behavioural counselling sessions) are likely to be most effective [22]. However, the use of cessation support within lung cancer screening programmes varies widely across countries and there is a need for clear guidelines for smoking cessation provision in lung cancer screening programmes, tailored for country-specific contexts.

The inclusion of complementary non-imaging-based tests is considered to be an important addition for refined identification of high-risk individuals. An example of such a study is the Italian BioMILD lung cancer screening trial, which evaluated the utility of combining a plasma 24-microRNA signature classifier and LDCT to define the individual risk and personalise screening strategies [23]. The molecular signature predicted an increased risk of lung cancer with a hazard ratio of 4:4 for lung cancer incidence and of 8:1 for lung cancer mortality (both significantly increased) at 7 years of follow-up. Another biomarker-based study investigated pre-diagnostic proteomics measurements using a 22-protein panel compared with existing prediction tools, including the commercial autoantibody-based Early Cancer Detection Test (EarlyCDT)-Lung and the PLCOm2012 [19]. The circulating protein panel showed promising results and indeed outperformed both existing risk prediction methods. Both the above-mentioned studies are examples of how the Nordic lung cancer screening programmes could expand to also include non-imaging-based tests to further refine the predictive models. There are numerous other biomarker candidates [24], both in blood and sputum, for early detection of lung cancer, both with high sensitivities, high specificities or both. These biomarker candidates need to be further assessed in studies using adequate sample sizes, control groups with non-malignant conditions, and standardised cut-off levels for the different biomarkers.
